# Which clustering algorithm is better for predicting protein complexes?

**DOI:** 10.1186/1756-0500-4-549

**Published:** 2011-12-20

**Authors:** Charalampos N Moschopoulos, Georgios A Pavlopoulos, Ernesto Iacucci, Jan Aerts, Spiridon Likothanassis, Reinhard Schneider, Sophia Kossida

**Affiliations:** 1 Bioinformatics & Medical Informatics Team, Biomedical Research Foundation, Academy of Athens, Soranou Efessiou 4, 11527 Athens, Greece; 2 Department of Computer Engineering & Informatics, University of Patras, Rio, GR-26500 Patras, Greece; 3 Department of Computer Science and Biomedical Informatics, University of Central Greece, Papasiopoulou 2-4, 35100 Lamia, Greece; 4 ESAT-SCD/IBBT-K.U.Leuven Future Health Department, Katholieke Universiteit Leuven, Kasteelpark Arenberg 10, box 2446, 300, Leuven, Belgium; 5 Bioinformatics/Structural and Computational Biology, European Molecular Biology Laboratory, Meyerhofstrasse 1, 69117 Heidelberg, Germany; 6 Luxembourg Centre for Systems Biomedicine (LCSB), University of Luxembourg, Campus Limpertsberg, 162 A, avenue de la Faïencerie, 1511 Luxembourg, Germany

## Abstract

**Background:**

Protein-Protein interactions (PPI) play a key role in determining the outcome of most cellular processes. The correct identification and characterization of protein interactions and the networks, which they comprise, is critical for understanding the molecular mechanisms within the cell. Large-scale techniques such as pull down assays and tandem affinity purification are used in order to detect protein interactions in an organism. Today, relatively new high-throughput methods like yeast two hybrid, mass spectrometry, microarrays, and phage display are also used to reveal protein interaction networks.

**Results:**

In this paper we evaluated four different clustering algorithms using six different interaction datasets. We parameterized the MCL, Spectral, RNSC and Affinity Propagation algorithms and applied them to six PPI datasets produced experimentally by Yeast 2 Hybrid (Y2H) and Tandem Affinity Purification (TAP) methods. The predicted clusters, so called protein complexes, were then compared and benchmarked with already known complexes stored in published databases.

**Conclusions:**

While results may differ upon parameterization, the MCL and RNSC algorithms seem to be more promising and more accurate at predicting PPI complexes. Moreover, they predict more complexes than other reviewed algorithms in absolute numbers. On the other hand the spectral clustering algorithm achieves the highest valid prediction rate in our experiments. However, it is nearly always outperformed by both RNSC and MCL in terms of the geometrical accuracy while it generates the fewest valid clusters than any other reviewed algorithm. This article demonstrates various metrics to evaluate the accuracy of such predictions as they are presented in the text below. Supplementary material can be found at: http://www.bioacademy.gr/bioinformatics/projects/ppireview.htm

## Background

Proteins are the main actors responsible for virtually every function within a cell. While some proteins are characterized by a unique function, the majority of them operate in coordination with other proteins forming PPI networks to carry out processes in the cell. Such processes include cell cycle control, differentiation, protein folding, signaling, transcription, translation, post-translational modification and transportation. Trying to understand and predict protein functions at a systems level is neither a straightforward nor a trivial task. Due to such issues, which range from wet-lab technical challenges to the innate complexity of high dimensional data analysis, function prediction has become one of the most important and difficult challenges in current computational biology research.

Some of the most well known techniques to reveal information about the interaction of proteins are the pull down assays [1] and tandem affinity purification [2]. State of the art high-throughput methods such as yeast two hybrid systems--Y2H [3], mass spectrometry [4], microarrays [5] and phage display [6] are able to generate enormous datasets of PPIs with high quality of information. While the aforementioned techniques are valuable tools to capture the role of molecular functions at a systems level, their main drawback is that the resulting datasets are often incomplete and exhibit high false positive and false negative rates.

In addition to the direct experimental data, a wide range of large biological databases storing information about validated or predicted PPI data is also available. The Yeast Proteome Database--YPD [7], for example, combines protein-interaction and other data from the literature. A number of other important databases that curate protein and genetic interactions of yeast from the literature have been developed, including the Munich Information Center for Protein Sequences--MIPS database [8], the Molecular Interactions--MINT database [9] the IntAct database [10], the Database of Interacting Proteins--DIP [11], the Biomolecular Interaction Network Database--BIND [12], and the BioGRID database [13]. A number of public repositories for human PPIs are currently available, including the databases: BIND [12], DIP [11], IntAct [10], MINT [9] and MIPS [14]. There exist also organism specific databases such as the Human Protein Reference Database--HPRD [15] or the HPID [16] for human or DroID [17] for Drosophila.

Proteins can either act individually or as a part of bigger system to perform an intricate process in the cell. Thus, proteins often collaborate and form stable associations, termed protein complexes [4,18,19]. In a larger network consisting of nodes (proteins) and edges (PPI interactions), a protein complex corresponds to a dense subgraph (aggregation of highly interconnected vertices) or even a clique. Identification of such complexes in PPI graphs is an important challenge and can be of valuable help in understanding the cell functions. Computational methods such as MCODE [20], jClust [21], Clique [22], LCMA [23], DPClus [24], CMC [25], SCAN [26], Cfinder [27], GIBA [28] or PCP [29] are graph-based algorithms that use graph theory to detect highly connected subnetworks. DECAFF [30], SWEMODE [31] or STM [32] have been developed to predict protein complexes incorporating graph annotations, whereas others like DMSP [33], GFA [34] and MATISSE [35] take also the gene expression data into account. A very useful review article that describes and compares the aforementioned techniques can be found in [36].

In this study, we to go one step further than [36] and benchmark four different clustering algorithms against six different datasets not covered in [36] to evaluate how well widely used clustering algorithms like the aforementioned can predict protein complexes from PPI data. The algorithms which we tested include the MCL [37], RNSC [38], Affinity Propagation [39] and Spectral clustering [40]. All these algorithms assign each protein of the PPI graph to a cluster. The datasets used are: Tong [41], Krogan [42], Gavin 2002 [4], Gavin 2006 [19], DIP [11] and the MIPS [43]. To evaluate the accuracy and the percentage of valid predictions of the algorithms against the specific datasets we used the MIPS [8] and the set of complexes derived from [44] as benchmarks.

## Methods

### Data preparation integration

In this section, we give a short description of the datasets that were used in this study. All of the current datasets hold information about unweighted PPI associations.

#### Tong dataset [41]

This network consists of 7430 edges and 2262 vertices. A genetic interaction network was mapped by crossing mutations in several genes into a set of viable gene yeast deletion mutants scoring the double mutant progeny for fitness defects. The interactions of this network were produced by predicting the functions of the interactive elements. These elements are often produced by bringing together functionally related genes, components, or proteins that belong to the same pathway. The genetic network exhibited dense local neighbourhoods.

#### Krogan dataset [42]

This dataset consists of 7088 edges and 2675 vertices and contains different tagged proteins of the yeast *Saccharomyces cerevisiae *organism. In the original article, the MCL [37] algorithm was used to cluster and organize the proteins into several groups.

#### Gavin 2002 [4] and Gavin 2006 [19] datasets

Gavin 2002 [4] dataset consists of 3210 edges and 1352 vertices, whereas Gavin 2006 [19] consists of 6531 edges and 1430 vertices. In the first dataset, large-scale tandem affinity purification and mass spectrometry were used to characterize multi-protein complexes in *Saccharomyces cerevisiae*. Extending this information to the human genome, this dataset provides an outline of the eukaryotic proteome as a network of protein complexes. Using the whole network, we try to see how successfully the various methods detect the network complexes. The second dataset contains the first genome-wide screen for complexes in yeast.

#### DIP dataset [11]

The Database of Interacting Proteins (*DIP*) stores experimentally validated protein-protein interactions. We used this database to isolate a network of 17491 edges and 4934 vertices. We included this dataset for our experiments because, aside from protein-protein interactions, the DIP database provides abundant annotations to allow deeper understand of the protein functions.

#### MIPS dataset [43]

The Munich Information Center for Protein Sequences (MIPS) provides resources mainly related to genome information. Most of the databases that store evidences about a diversity of genomes of distinctive organisms are manually curated. In addition, 400 genomes, which are annotated automatically, are also integrated. In this case study, the network consists of 12526 edges and 4554 vertices given by the MIPS database.

It should be noted that our experiments for testing clustering algorithms are strictly limited to unweighted PPI datasets. Other kinds of protein interactions (Enzyme-Inhibitor and antigen-antibody) concern specific subcategories of interactions that can not form large scale networks as PPI datasets do. For example, the two datasets of antigen-antibody complexes presented in [45] could not be used in our survey as they contain very few data points and are derived from different organisms. It is notable that RNSC algorithm does not take edge weights into account in its function.

### Clustering techniques

The algorithms, used here, to predict protein complexes include the MCL [37], RNSC [38], affinity propagation [39] and spectral clustering [40]. The decision to include these algorithms in our setting was reached due to the fact that they are widely used but also because they perfectly complement the study carried out in [36]. We wish to make clear that we did not evaluate any algorithms for clique detections since such algorithms specialize in detecting fully connected sub-areas of the network. Such a comparison would be unfair since clustering techniques tend to predict a much higher number of complexes, and often detect the cliques. For the MCL and RNSC algorithms, the original versions were used. These can be found at: http://micans.org/mcl/ and http://rsat.bigre.ulb.ac.be/rsat/index_neat.html respectively. For the spectral clustering and affinity propagation algorithms we used the versions incorporated within the jClust [21] application. Below we give some information about the main concept that the algorithms are based on.

#### MCL [37]

The MCL algorithm is a fast and scalable unsupervised clustering algorithm. It is one of the most widely used algorithms and is it is based on simulating stochastic flows in networks. The MCL algorithm can detect cluster structures in graphs by taking advantage of a mathematical bootstrapping procedure. The process is trying to perform random walks through a graph and deterministically compute their probabilities to find the best paths. It does so by using stochastic Markov matrices. The algorithm works by alternating the inflation parameter, which iteratively calculate the set of transition probabilities. The inflation operator implements a stochastic matrix transformation to emphasize larger probabilities and deemphasize smaller ones.

#### RNSC [38]

The RNSC algorithm initially searches for a low cost clustering by initializing a random clustering. It then iteratively assigns nodes to different clusters randomly to improve the clustering cost. In order to avoid local minima, RNSC makes diversification note transfers and performs multiple experiments. Furthermore, it maintains dynamic data structures to prevent exploring back a previously visited partitioning. The functionality of the RNSC algorithm depends on various parameters needed for the Tabu search step (Tabu length and Tabu list tolerance), as well as the terminating criteria (naïve stopping tolerance and scaled stopping tolerance) and other (maximal number of clusters, diversification frequency and shuffling diversification length). Further analysis concerning these parameters can be found in [38].

#### Affinity Propagation [39]

Affinity propagation is an unsupervised algorithm and thus the number of clusters are automatically calculated. The idea behind this algorithm is to find sub-paths, which allow easy message exchanges between nodes. It takes as input a similarity matrix, which keeps the distances between all possible pairs of data points whereas it initially considers all data points as potential "exemplars". In later steps, real-valued messages are exchanged between the nodes until a set of exemplars and corresponding clusters emerges with high quality. The main parameter of this algorithm is the 'preference', which controls how many data points are selected as exemplars.

#### Spectral clustering [40]

This algorithm tries to detect clusters in a graph, where nodes are connected with highly-similarity. The algorithm also tries to find connections between such areas that should be weak, constituted by edges of low similarity. The aim is to identify highly connected clusters and, at a later stage, filter the inter edges within the cluster. The only parameter required is the user-defined number of clusters.

### Evaluation

To evaluate the algorithms against specific datasets that already contain information about recorded protein complexes we used the MIPS [8] database and the set of complexes derived from [44] (denoted as BT_409) as benchmarks. Concerning the MIPS protein complexes dataset, we observed that often information is stored in a hierarchical structure. To avoid redundancies, the parent complexes were discarded and the sub-complexes were retained. The final evaluation dataset comprises 220 complexes. The BT_409 dataset similarly contains 409 complexes and is composed by applying a bootstrapping strategy on tandem affinity purification data. It and has been also used as a benchmark dataset in other studies such as [36].

To determine whether a sub-graph represents a protein complex or not, we compared each derived cluster against every recorded protein complex in the MIPS or BT_409 dataset. We used the same evaluation metric adopted in [20], called the ***geometric similarity index***. This method considers a predicted complex as valid if $\frac{I^{2}}{A*B}>0,2$, where ***I ***is the number of common proteins, ***A ***the number of proteins in the predicted complex and ***B ***the number of proteins in the recorded complex. Finally, we kept the highest geometric similarity index (score), which a predicted cluster achieved over the recorded ones.

Moreover, 3 different matching statistical metrics, that were presented in [46] and [36], were used in the evaluation process of the tested algorithms. These are ***sensitivity *(Sn)**, ***Positive Predictive Value *(PPV) **and ***Geometrical Accuracy *(Acc_g)**. The mathematical formulas of the above statistical measurements are given below. Given *n *benchmark complexes and *m *predicted complexes, let *T*_*ij *_denote the number of proteins in common between *i*^th ^benchmark complex and *j*^th ^predicted complex. *Sn, PPV *and *Acc_g *are then defined as follows:

(1) $$Sn=\frac{\overset{n}{\underset{i=1}{\sum }}\max_{j}\left\{T_{ij}\right\}}{\overset{n}{\underset{i=1}{\sum }}N_{i}},PPV=\frac{\overset{m}{\underset{j=1}{\sum }}\max_{i}\left\{T_{ij}\right\}}{\overset{m}{\underset{j=1}{\sum }}T_{j}},Acc\text{\_}g=\sqrt{Sn*PPV}$$

*N*_*i *_indicates the number of proteins belonging to recorded complex *i *and *T*_*j *_indicates the total number of members of *j *predicted complex assigned to all benchmark complexes. These metrics are widely used to measure the correspondence between the result of a classification and a reference and to provide an overview of how accurately the clustering techniques can detect the protein complexes from PPI data.

The aforementioned metrics come with their strengths and their limitations. In the case of sensitivity (*Sn*), if a method predicts very big complexes with many proteins, the *Sn *score will tend to have very high values. The *PPV *value on the other hand, does not evaluate overlapping clusters properly. In addition, all of the evaluation metrics described above assume that a complete set of real protein complexes is available, but this does not necessarily corresponds to the real experimental data.

Finally, two more metrics were used for our evaluation procedure. These are the *absolute number of predictions *and the *mean score of valid predicted complexes*. The absolute number of predicted clusters is a metric that measures the efficiency of the tested algorithms to identify as many protein complexes as possible in a PPI graph. This metric varies as the datasets tested vary regarding their density and the number of protein complexes which they contain. The absolute number of valid predictions is represented as ***a/b ***where ***a ***is the number of valid predicted complexes and ***b ***the total number of the derived clusters. The mean score of valid predicted clusters indicates the mean geometric similarity index of the predicted clusters that surpass the threshold of 0.2. This metric is used in order to measure how well a recorded complex is predicted by the algorithms tested.

## Results

Extensive experiments to compare the aforementioned techniques were performed. The comparison of these methods is less biased than in other reviews so far such as [36,46] where different types of algorithms based on different clustering approaches are compared with each other, which can be misleading.

During the first step of our experiment, the four algorithms are applied on the six aforementioned datasets and the resulting clusters are compared respectively. During the second step, the results of the tested algorithms are filtered according to the methodology introduced in [47]. A thorough analysis was performed to show the consequence of the post-clustering filter parameters on each of the tested algorithms and how they can affect the final results. For our experiments, a wide range of values and parameters for the algorithms parameters was essayed. However, it must be noted that there is no strict way to set the algorithms parameters in order to produce the optimal results for every dataset. For instance, the MCL algorithm produces higher valid prediction rates, which means that the percentage of valid predicted clusters to total in the MCL results is higher, when the inflation parameter is set to 1.8 and higher accuracy rates when it is set to 2. In order to compare MCL results with other algorithms we used those produced by MCL when the inflation parameter is set to 1.8. More information concerning the MCL algorithm behavior across different values of the inflation parameter, can be found in Additional File 1. For the affinity propagation algorithm, we used the scripts (preferenceRange.m and apclusterK.m) which are available at [48]. In order to determine the parameters of the algorithm we used the eigengap heuristic [49] which searches the structure of the network in order to automatically elucidate the number of clusters in the network. Finally, for the RNSC algorithm we used the values presented in [36,46] due to the numerous parameters that this algorithm uses.

**Figure 1:**
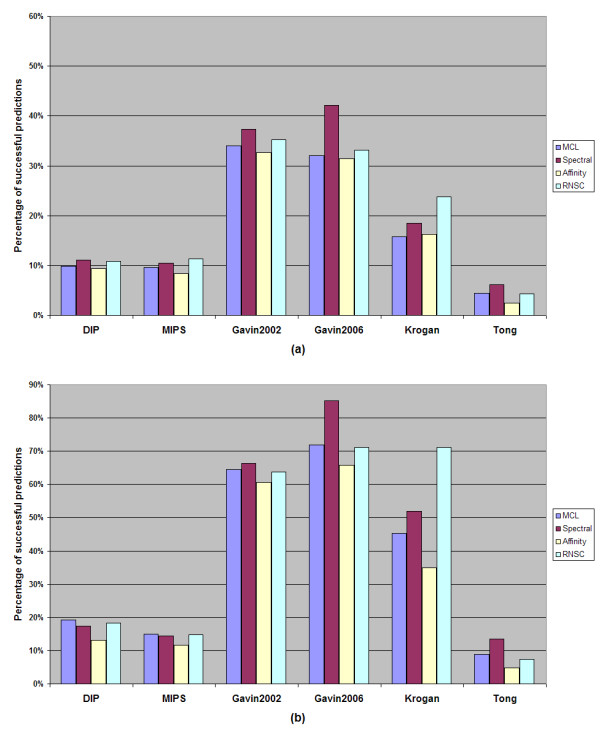
**The percentage of valid predictions of every algorithm on each dataset**. **a **Using the MIPS recorded protein complexes as evaluation set. **b **Using BT_409 as evaluation set

**Figure 2:**
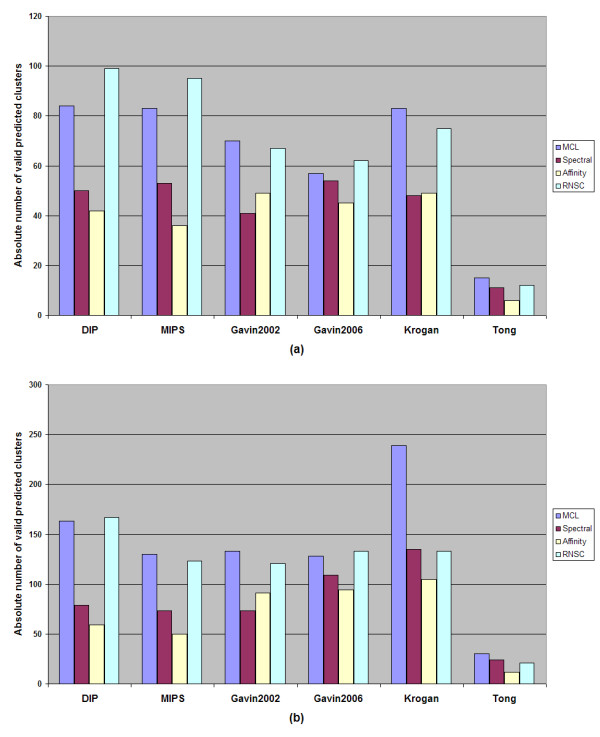
**The absolute number of each time valid predictions on each dataset**. **a **Using the MIPS recorded protein complexes as evaluation set. **b **Using BT_409 as evaluation set

More detailed information about the results of our experiments, presented in this manuscript, is additionally provided as supplementary material (Additional File 1). Figures 1 and 2 show the percentage of valid predictions of every algorithm for each dataset and the absolute number of valid predictions, which indicates the number of the derived clusters that overcome the threshold of 0.2 of the geometric similarity index metric compared to the recorded MIPS complexes or BT_409 dataset.

**Figure 3:**
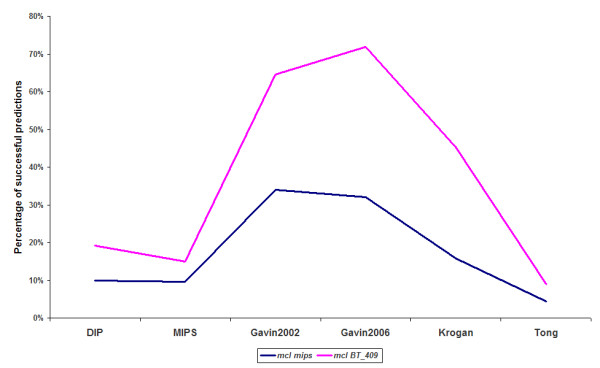
**The percentage of valid predictions of the MCL algorithm on every dataset**. X axis: the datasets, Y axis: The valid predictions

A trade-off between the absolute number and the percentage of valid predictions is apparent for the spectral clustering algorithm. Although it surpasses, in most cases, all the other algorithmic techniques in the percentage of valid predictions, it does not generate as many valid predicted clusters as the MCL and the RNSC do. According to Figures 1 and 2, the MCL and RNSC algorithms achieve the best prediction rates in several cases (two out of six in Figure 1a and three out of six in Figure 2a) and the best performances regarding the absolute number of valid predictions. The tested algorithms produce more valid clusters when the BT_409 evaluation set is used compared to the MIPS dataset. This is expected as BT_409 contains almost the double number of protein complexes compared to the MIPS golden standard (409 against 220 respectively). As a result, the rate of valid prediction is higher for all algorithms when the BT_409 evaluation set is used. For instance, in Figure 3, the percentage of valid predictions of the MCL algorithm for each dataset is shown. In all cases, the MCL algorithm achieves higher rates when the BT_409 evaluation set is used. In the Gavin 2002, Gavin 2006, and Krogan datasets, the difference between the two evaluation sets is very obvious, while in the MIPS and Tong datasets it is minimized.

In order to check the robustness of the tested algorithms against noise, we performed experiments with 3 altered datasets presented in [46]. The results are presented in Additional File 1, Table S5, and each dataset is noted as *complexes_rm_i_ad_j*, where *i *and *j *indicate the percentage of deleted and added edges respectively to the PPI graph formed by the collection of MIPS recorded protein complexes.

**Figure 4:**
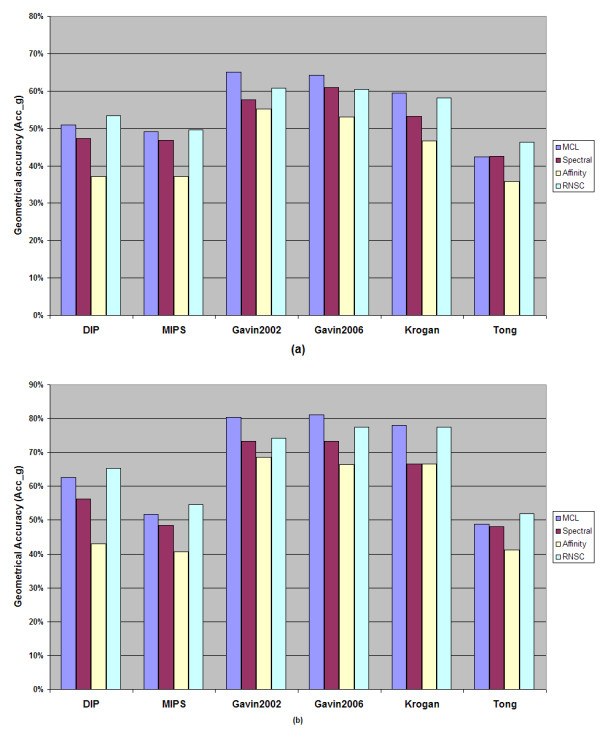
**The performance of the algorithms concerning Acc_g metric on each dataset**. **a **Using the MIPS recorded protein complexes as evaluation set. **b **Using BT_409 as evaluation set

Figure 4, shows the performance of the algorithms according to the geometrical accuracy metric. As we mentioned before, the geometrical accuracy offers a better insight concerning the quality of the results of each algorithm as its value depends on the *Sensitivity *(Sn) and *Positive Predictive Value *(PPV) metrics.

## Discussion

We performed extensive experiments using four different algorithmic strategies to detect protein complexes in six PPI networks. For the evaluation process, two different evaluations sets were used. These were a) the golden standard of MIPS recorded protein complexes and b) the BT_409 dataset.

In the cases where the MIPS dataset was used for evaluation, the spectral clustering algorithm achieved the highest performance with respect to the percentage of valid predictions compared to the other algorithms. On the other hand, the RNSC and MCL algorithms were the methods that clearly generated the most valid clusters. Regarding the BT_409 evaluation set (see Figure 1b), the RNSC algorithm performs as well as the spectral clustering whereas in some cases it surpasses it. The MCL and RNSC algorithms performed best according to Acc_g metric. These algorithms produced high quality results as the derived clusters match more accurately the recorded protein complexes in MIPS and BT_409 evaluation sets.

According to our results, in line with [36], the RNSC algorithm behaved better than the other algorithms. It produced many valid clusters whereas in almost all datasets it was ranked as the second best algorithm with respect to the percentage of the valid predictions (in some cases surpassed spectral clustering algorithm) as this can be seen in Figure 1. It should be noted that, if the affinity propagation algorithm would be excluded, the difference concerning the geometrical accuracy between the RNSC and the other two algorithms (MCL and spectral clustering) would not be as big as the difference between the affinity propagation algorithm and the others (MCL and spectral clustering).

The MCL and RNSC algorithms performed similarly but in most cases, RNSC surpassed the MCL algorithm by giving higher valid prediction rate. However, both algorithms achieved high accuracy and high absolute number of valid predicted clusters in all datasets compared to the rest of the algorithms. On the other hand, the MCL algorithm performed better in most cases comparing to tother methods with respect to the mean score of valid predicted complexes which shows a mean geometric similarity index of the predicted clusters that surpass the threshold of 0.2. This metric was used in order to measure how well a recorded complex is predicted by the algorithms tested.

The Affinity propagation algorithm seemed to have lower performance than the rest of the tested algorithms. The number of iterations was set to the dataset size. A direct and more generic comparison between the Affinity propagation and the MCL algorithms can be found at [50].

Finally, regarding the robustness of the algorithms against noise, the spectral clustering and RNSC algorithms performed best as it can be seen in Table S5 of the Additional File 1. More specifically, spectral clustering algorithm achieved the highest percentage of successful predictions while the RNSC algorithm achieved the highest performance with respect to the geometrical accuracy metric and the absolute number of valid predicted clusters. The affinity propagation algorithm seems to be the most sensitive to the noisiness of the data. On the other hand, the MCL algorithm can be considered as the most stable one and performs best when the noise in the data becomes inordinate.

In the second phase of the performed experiments, the results of the tested algorithms were filtered according to the methodology introduced in [47]. A total number of 17 different combinations of the post-cluster filtering process were applied to the algorithms results, forming a stringent or less stringent filter. The range of parameters for the four methods that constitute the applied filter is shown in Table 1. Choosing a single parameter value out of the proposed range would be meaningless because the parameter method would become either too rigorous and it would produce very few clusters (if it was higher than the proposed maximum) or it would add noise to the final data (if it was lower than the proposed minimum).

**Table 1:** Method parameters range of values

Parameter	Value range
Density parameter	[0.5, 0.7]

Best neighbor parameter	[0.5, 0.75]

Cutting edge parameter	[0.5, 0.75]

Haircut parameter	[2,3] only integer values

All the results of our experiments with the varying post-cluster filtering parameters are presented in Additional file 2 and Additional file 3. As expected, the density method has the biggest affect concerning the number of the final clusters of each algorithm than any other filtering method. The higher the value of this parameter, the fewer the clusters, which were generated by the tested algorithms that could pass the filter, are. The Gavin 2006 and Krogan datasets are the best examples for the algorithm to be applied on, since they generated more clusters comparing to any other dataset. On the other hand, the Tong dataset, due to its sparseness, does not help the algorithms to achieve high prediction rate or absolute number of valid clusters. When the filtering step is added, all of the algorithms produce extremely few clusters but with a higher probability of these clusters to be valid.

Going one step further, we compared the five best performances of each algorithm combined with the post-cluster filtering process which also produced more than ten final and more than three valid clusters. Had this not been carried out, the comparison would be biased because, for an example, one algorithm would produce only one valid cluster, which would have 100% score according to the geometrical accuracy metric. Only in one case where affinity propagation algorithm was evaluated against the MIPS golden standard dataset, there were no results that could satisfy the above prerequisites. All of the results can be found in Additional file 4: where the geometrical accuracy and the absolute number of valid predicted clusters are plotted.

The first conclusion, which can be derived, is that all algorithms achieved much higher values for geometrical accuracy metric. Regarding the experiments performed which use MIPS golden standard as evaluation set; in most of the cases the affinity propagation algorithm achieves the highest mean geometrical accuracy. However, this can be explained by the fact that the best results achieved by affinity propagation algorithm produce fewer valid clusters than any other algorithm. On the other hand, the RNSC algorithm seems to achieve poorer performance for geometrical accuracy but, together with MCL algorithm, they produce the most valid predicted clusters.

When the evaluation set used is the BT_409 dataset, the spectral clustering and the RNSC algorithms achieve the best performance based on geometrical accuracy metric. Concerning the absolute number of valid predictions, the MCL and RNSC algorithms produced the highest scores. Notably, all algorithms achieved higher accuracy values for BT_409 dataset while their final valid clusters where approximately equal to those produced when the MIPS golden standard dataset was used.

It could be said that the post-cluster filtering process eliminated the differences between the algorithms regarding the geometrical accuracy metric. However, in many cases, spectral clustering and affinity propagation algorithms produced very few clusters and their results could not be exploited. Finally, it seems that all algorithms produced better results when the filter parameters where set according to Table 2.

**Table 2:** Filter method parameters values, which generally produced good results

Method	Value range
Density	0.5

Best neighbor	0.65

Cutting edge	0.75

Haircut	2

## Conclusion

Six PPI network datasets were subjected to four different algorithmic strategies. The motivation behind this approach is to benchmark the clustering techniques and measure their prediction accuracy to detect protein complexes. For the evaluation process, two different evaluations sets were used. It is notable that we evaluated algorithms that share similar concepts to cluster networks. After essaying various parameters for the aforementioned algorithms we found that the RNSC and MCL algorithms are more accurate in predicting PPI complexes as they outperformed the other algorithms concerning the geometrical accuracy metric and the mean score of valid predicted complexes. In contrast, the spectral clustering algorithm achieves the highest valid prediction rate in our experiments but fails to surpass the RNSC and MCL algorithms concerning the geometrical accuracy metric and the absolute number of the valid predicted clusters.

## Competing interests

The authors declare that they have no competing interests.

## Authors' contributions

CNM was behind the experimental part. He provided the statistical analysis and the evaluation of the clustering algorithms as presented in the Results section. He analyzed the data and demonstrated how each clustering algorithm behaves for every PPI dataset. GAP was responsible for collecting, running and evaluating the clustering algorithms. GAP together with CNM wrote scripts to perform a great number of experiments simultaneously. Scripts were also produced to compare the predicted results with already known complexes stored in databases to show how reliable the prediction of each algorithm is. EI provided critical assessment and participated in the statistical analysis of the methods presented. JA evaluated the results. SL supervised the experimental procedure and RS provided the computational power at EMBL for large-scale analysis. SK was the main supervisor of the project. All of the aforementioned authors wrote parts of the manuscript. All of the authors have read and approved the manuscript.

## Supplementary Material

Additional file 1 **Supplementary tables**. Summary of experimental results using MIPS protein complexes as evaluation dataset Click here for file

Additional file 2 **Experimental results of each algorithm combined with the filter process, using MIPS protein complexes as evaluation dataset**. Click here for file

Additional file 3 **Experimental results of each algorithm combined with the filter process, using BT_409 protein complexes as evaluation dataset**. Click here for file

Additional file 4 **Figure S1**. The performance of the five best performances of each algorithm combined with the filter process concerning the ACC_g metric on each dataset: (**a**) when the MIPS golden standard is used for evaluation, (**b**) when the BT_409 dataset is used for evaluation. Click here for file
