# Supplementary material for: Which clustering algorithm is better for predicting protein complexes?
Source: BMC Res Notes. 2011 Dec 20;4:549. doi: 10.1186/1756-0500-4-549 (PMC3267700; doi:10.1186/1756-0500-4-549)
Supplement: Additional file 1 — Supplementary tables. Summary of experimental results using MIPS protein complexes as evaluation dataset [file 1756-0500-4-549-S1.PDF]

Table S1. Summary of experimental results using MIPS protein complexes as evaluation dataset.

Algorithms	Percentage of successful prediction	Absolute number of predictions	Mean Score of valid predicted complexes	Mean predicted size of valid clusters	Sn	PPV	Acc_g
**DIP dataset**							
**MCL**	9,91%	84/848	0,468	6,988	48,96%	52,97%	50,93%
**Spectral**	11,04%	50/453	0,441	7,801	66,84%	33,46%	47,29%
**Affinity**	9,40%	42/447	0,422	6,690	37,39%	36,73%	37,06%
**RNSC**	10,83%	99/914	0,483	6,717	49,64%	57,40%	53,38%
**MIPS dataset**							
**MCL**	9,57%	83/867	0,482	5,373	45,69%	52,67%	49,06%
**Spectral**	10,47%	53/506	0,457	5,962	58,68%	37,37%	46,83%
**Affinity**	8,35%	36/431	0,426	5,833	38,47%	35,97%	37,20%
**RNSC**	11,35%	95/837	0,443	5,442	43,83%	56,25%	49,65%
**Gavin_2002 dataset**							
**MCL**	33,98%	70/206	0,579	8,214	74,26%	56,97%	65,04%
**Spectral**	37,27%	41/110	0,536	8,951	75,84%	43,85%	57,67%
**Affinity**	32,67%	49/150	0,534	7,510	64,47%	47,25%	55,19%
**RNSC**	35,26%	67/190	0,475	6,925	67,78%	54,51%	60,79%
**Gavin_2006 dataset**							
**MCL**	32,02%	57/178	0,531	10,053	76,42%	53,97%	64,22%
**Spectral**	42,19%	54/128	0,488	8,333	75,03%	49,59%	61,00%
**Affinity**	31,47%	45/143	0,511	7,533	61,80%	45,61%	53,09%
**RNSC**	33,16%	62/187	0,482	8,323	65,32%	55,99%	60,48%
**Krogan dataset**							
**MCL**	15,72%	83/528	0,520	7,506	63,07%	56,09%	59,48%
**Spectral**	18,46%	48/260	0,500	9,188	70,45%	40,30%	53,28%
**Affinity**	16,28%	49/301	0,506	6,592	52,01%	41,71%	46,58%
**RNSC**	23,73%	75/316	0,424	9,000	61,84%	54,59%	58,10%
**Tong Dataset**							
**MCL**	4,44%	15/338	0,357	4,733	43,95%	40,89%	42,39%
**Spectral**	6,15%	11/179	0,405	3,545	55,83%	32,32%	42,48%
**Affinity**	2,44%	6/246	0,317	3,333	41,14%	31,29%	35,88%
**RNSC**	4,26%	12/282	0,386	6,000	47,87%	44,72%	46,27%

Table S2. Summary of experimental results using BT_409 protein complexes collection as evaluation dataset.

Algorithms	Percentage of successful prediction	Absolute number of predictions	Mean Score of valid predicted complexes	Mean predicted size of valid clusters	Sn	PPV	Acc_g
**DIP dataset**							
**MCL**	19,22%	163/848	0,421	5,785	59,94%	65,29%	62,56%
**Spectral**	17,44%	79/453	0,420	9,114	73,16%	43,26%	56,26%
**Affinity**	13,20%	59/447	0,413	5,847	45,50%	40,72%	43,04%
**RNSC**	18,27%	167/914	0,453	6,234	58,52%	72,94%	65,33%
**MIPS dataset**							
**MCL**	14,99%	130/867	0,397	4,200	46,14%	57,98%	51,72%
**Spectral**	14,43%	73/506	0,408	4,315	58,63%	40,03%	48,45%
**Affinity**	11,60%	50/431	0,378	5,060	43,91%	37,56%	40,61%
**RNSC**	14,70%	123/837	0,394	4,675	47,06%	63,27%	54,57%
**Gavin_2002 dataset**							
**MCL**	64,56%	133/206	0,617	7,850	86,66%	74,63%	80,42%
**Spectral**	66,36%	73/110	0,603	9,370	87,25%	61,66%	73,35%
**Affinity**	60,67%	91/150	0,531	7,462	72,58%	60,69%	66,37%
**RNSC**	63,68%	121/190	0,535	6,512	74,95%	73,57%	74,26%
**Gavin_2006 dataset**							
**MCL**	71,91%	128/178	0,615	8,914	92,60%	71,08%	81,13%
**Spectral**	85,16%	109/128	0,572	9,330	86,82%	61,91%	73,32%
**Affinity**	65,73%	94/143	0,583	7,617	75,93%	58,35%	66,56%
**RNSC**	71,12%	133/187	0,530	7,647	81,64%	73,53%	77,48%
**Krogan dataset**							
**MCL**	45,27%	239/528	0,601	5,841	77,12%	79,12%	78,11%
**Spectral**	51,92%	135/260	0,558	6,904	81,04%	54,69%	66,57%
**Affinity**	34,88%	105/301	0,544	5,838	60,76%	50,88%	55,60%
**RNSC**	71,12%	133/187	0,530	7,647	81,64%	73,53%	77,48%
**Tong Dataset**							
**MCL**	8,88%	30/338	0,373	5,833	53,77%	44,37%	48,84%
**Spectral**	13,41%	24/179	0,351	4,333	66,31%	34,77%	48,02%
**Affinity**	4,88%	12/246	0,355	3,167	51,20%	33,21%	41,24%
**RNSC**	7,45%	21/282	0,414	6,524	57,37%	46,92%	51,88%

Table S3. MCL algorithm behavior depending on the inflation parameter, using MIPS protein complexes as evaluation dataset.

Algorithms	Percentage of successful prediction	Absolute number of predictions	Mean Score of valid predicted complexes	Mean predicted size of valid clusters	Sn	PPV	Acc_g
**DIP dataset**							
**1.8**	9,91%	84/848	0,468	6,988	48,96%	52,97%	50,93%
**2**	10,05%	101/1005	0,449	6,000	46,90%	56,98%	51,70%
**2.2**	9,04%	105/1161	0,454	4,952	45,12%	59,15%	51,66%
**MIPS dataset**							
**1.8**	9,57%	83/867	0,482	5,373	45,69%	52,67%	49,06%
**2**	9,22%	93/1009	0,487	4,559	42,96%	56,74%	49,37%
**2.2**	9,07%	102/1125	0,501	4,049	41,76%	58,31%	49,35%
**Gavin_2002 dataset**							
**1.8**	33,98%	70/206	0,579	8,214	74,26%	56,97%	65,04%
**2**	29,79%	70/235	0,584	7,671	70,69%	58,50%	64,31%
**2.2**	29,44%	73/248	0,591	6,890	67,71%	59,25%	63,34%
**Gavin_2006 dataset**							
**1.8**	32,02%	57/178	0,531	10,053	76,42%	53,97%	64,22%
**2**	30,92%	64/207	0,531	9,625	74,60%	55,58%	64,39%
**2.2**	29,69%	68/229	0,548	8,044	72,84%	57,53%	64,73%
**Krogan dataset**							
**1.8**	15,72%	83/528	0,520	7,506	63,07%	56,09%	59,48%
**2**	14,53%	86/592	0,523	7,186	60,43%	57,05%	58,72%
**2.2**	14,80%	94/635	0,508	6,468	59,05%	58,48%	58,76%
**Tong Dataset**							
**1.8**	4,44%	15/338	0,357	4,733	43,95%	40,89%	42,39%
**2**	4,89%	18/368	0,346	4,389	43,18%	43,18%	43,18%
**2.2**	4,81%	19/395	0,344	4,263	42,21%	44,76%	43,47%

Table S4. MCL algorithm behavior depending on the inflation parameter, using BT_409 protein complexes collection as evaluation dataset.

Algorithms	Percentage of successful prediction	Absolute number of predictions	Mean Score of valid predicted complexes	Mean predicted size of valid clusters	Sn	PPV	Acc_g
**DIP dataset**							
**1.8**	19,22%	163/848	0,421	5,785	59,94%	65,29%	62,56%
**2**	18,71%	188/1005	0,427	4,920	56,97%	71,04%	63,62%
**2.2**	18,35%	213/1161	0,425	4,291	55,71%	75,26%	64,75%
**MIPS dataset**							
**1.8**	14,99%	130/867	0,397	4,200	46,14%	57,98%	51,72%
**2**	14,97%	151/1009	0,388	3,748	45,23%	63,37%	53,54%
**2.2**	14,67%	165/1125	0,411	3,412	45,75%	66,67%	55,23%
**Gavin_2002 dataset**							
**1.8**	64,56%	133/206	0,617	7,850	86,66%	74,63%	80,42%
**2**	58,72%	138/235	0,610	6,790	81,65%	76,59%	79,08%
**2.2**	58,06%	144/248	0,599	6,410	78,93%	77,43%	78,18%
**Gavin_2006 dataset**							
**1.8**	71,91%	128/178	0,615	8,914	92,60%	71,08%	81,13%
**2**	71,50%	148/207	0,600	7,764	92,28%	75,11%	83,25%
**2.2**	69,43%	159/229	0,592	7,069	88,98%	76,99%	82,77%
**Krogan dataset**							
**1.8**	45,27%	239/528	0,601	5,841	77,12%	79,12%	78,11%
**2**	43,41%	257/592	0,593	5,191	73,51%	81,01%	77,17%
**2.2**	42,52%	270/635	0,591	4,781	71,96%	83,52%	77,52%
**Tong Dataset**							
**1.8**	8,88%	30/338	0,373	5,833	53,77%	44,37%	48,84%
**2**	8,15%	30/368	0,373	5,333	53,37%	46,28%	49,70%
**2.2**	7,85%	31/395	0,374	5,129	52,63%	47,84%	50,18%

Table S5. Summary of experimental results with altered datasets, using MIPS protein complexes as evaluation dataset.

Algorithms	Percentage of successful prediction	Absolute number of predictions	Mean Score of valid predicted complexes	Mean predicted size of valid clusters	Sn	PPV	Acc_g
**Complexes_rm_10_ad_10**							
**MCL**	79.57%	74/93	0.789	10.432	81.11%	51.05%	64.34%
**Spectral**	97.32%	109/112	0.760	9.624	89.76%	61.82%	74.49%
**Affinity**	88.76%	79/89	0.673	12.215	83.19%	55.10%	67.71%
**RNSC**	99.25%	133/134	0.935	8.098	95.09%	68.95%	80.97%
**Complexes_rm_20_ad_20**							
**MCL**	85.00%	68/80	0.745	10.353	83.32%	53.92%	67.02%
**Spectral**	92.93%	92/99	0.710	10.848	93.03%	58.34%	73.67%
**Affinity**	81.44%	79/97	0.528	12.127	71.17%	50.03%	59.67%
**RNSC**	97.06%	132/136	0.813	8.008	92.58%	67.19%	78.87%
**Complexes_rm_40_ad_40**							
**MCL**	74.47%	35/47	0.374	4.086	88.73%	60.21%	73.09%
**Spectral**	81.00%	81/100	0.590	11.074	81.25%	52.98%	65.61%
**Affinity**	50.57%	44/87	0.337	15.364	51.89%	37.20%	43.94%
**RNSC**	64.29%	117/182	0.612	7.692	77.31%	60.09%	68.16%
