# Supplementary material for: Which clustering algorithm is better for predicting protein complexes?
Source: BMC Res Notes. 2011 Dec 20;4:549. doi: 10.1186/1756-0500-4-549 (PMC3267700; doi:10.1186/1756-0500-4-549)
Supplement: Additional file 4 — Figure S1. The performance of the five best performances of each algorithm combined with the filter process concerning the ACC_g metric on each dataset: (a) when the MIPS golden standard is used for evaluation, (b) when the BT_409 dataset is used for evaluation. [file 1756-0500-4-549-S4.PDF]

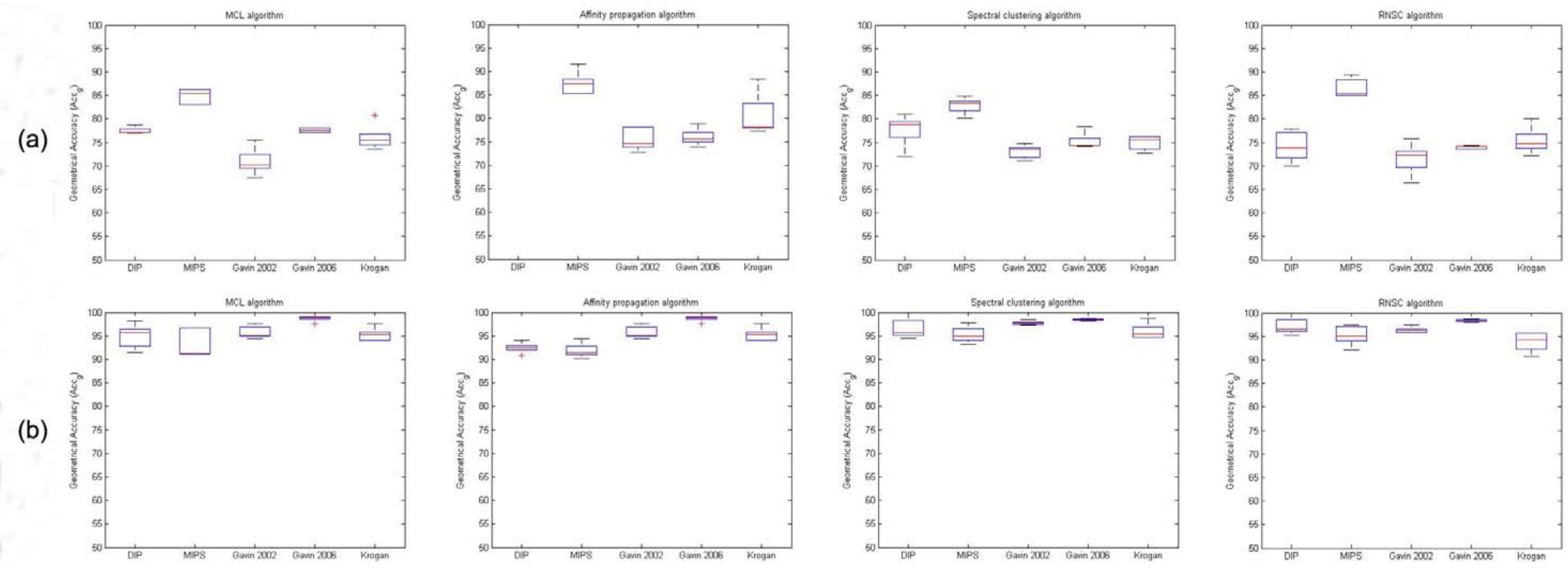


**Figure S1. The performance of the five best performances of each algorithm combined with the filter process concerning the ACC_g metric on each dataset: (a) when the MIPS golden standard is used for evaluation, (b) when the BT_409 dataset is used for evaluation.**

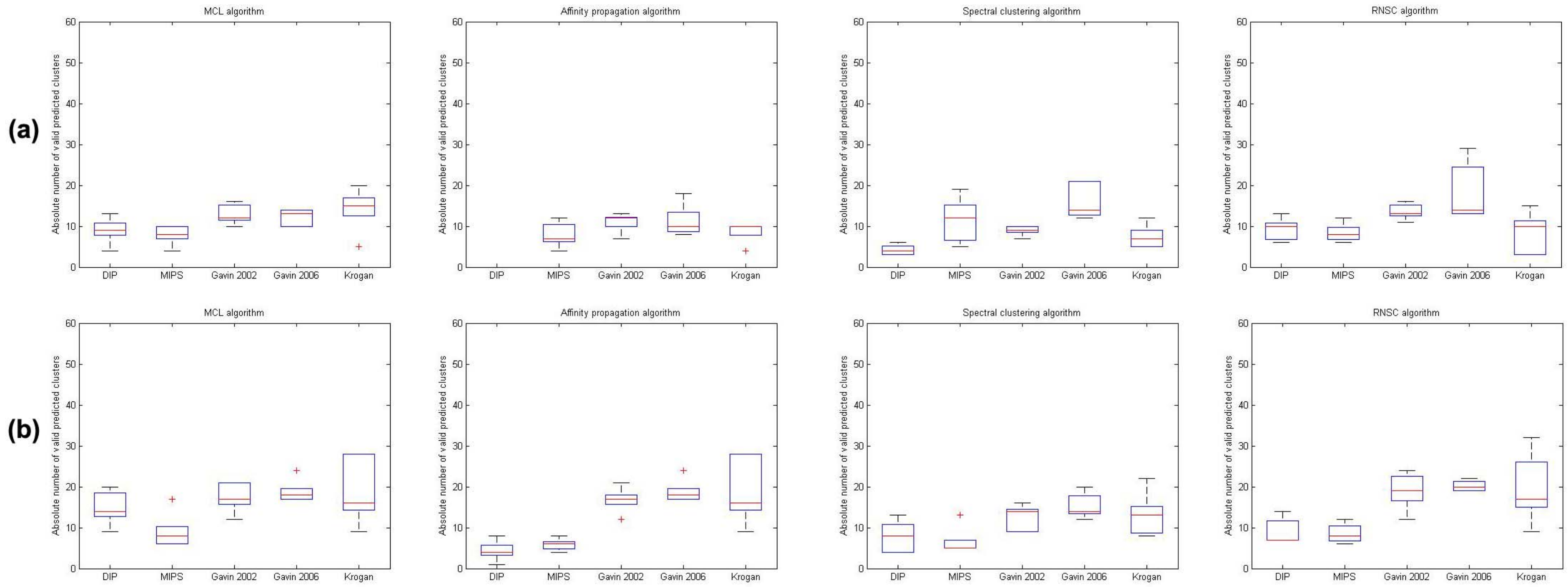


**Figure 2. The performance of the five best performances of each algorithm combined with the filter process concerning the absolute number of successful predictions on each dataset: (a) when the MIPS golden standard is used for evaluation, (b) when the BT_409 dataset is used for evaluation.**
